# High‐throughput quantitation of acetaldehyde and ethanol in mice using gas chromatography/mass spectrometry positive chemical ionization

**DOI:** 10.1111/acer.70126

**Published:** 2025-08-04

**Authors:** Yu‐Hong Lin, Cheng Chen, Shoupeng Wei, Guillot Adrien, Bryan Mackowiak, Hongna Pan, Yaojie Fu, Luca Maccioni, Tianyi Ren, Li Zhang, Joseph Hibbeln, Robert Pawlosky, Bin Gao

**Affiliations:** ^1^ Laboratory of Liver Diseases NIAAA/NIH Bethesda Maryland USA; ^2^ Laboratory of Integrative Neuroscience NIAAA/NIH Bethesda Maryland USA; ^3^ Department of Hepatology & Gastroenterology, Campus Virchow‐Klinikum and Campus Charité Mitte Charité ‐ Universitätsmedizin Berlin Berlin Germany; ^4^ University of Bristol Bristol UK; ^5^ Office of Scientific Director, DICBR NIAAA/NIH Bethesda Maryland USA

**Keywords:** direct PCA procedure, plasma, selected ion monitoring, serum, whole blood

## Abstract

**Background:**

Acetaldehyde, an immediate ethanol metabolite, mediates many ethanol‐induced behavioral effects and is both psychoactive and toxic to animals and humans. Monitoring the kinetics of acetaldehyde using rodent models of alcohol misuse is essential for understanding and managing ethanol‐associated diseases. However, quantitation of acetaldehyde in biological specimens after alcohol consumption has been challenging due to its high volatility, relatively low concentrations, and strong reactivity toward biochemical molecules. It was necessary to develop and establish an accurate and high‐throughput method to quantitate acetaldehyde and ethanol.

**Methods:**

Gas chromatography/mass spectrometry in positive chemical ionization mode coupled with a 111‐vial headspace autosampler was employed to quantitate acetaldehyde and ethanol using ^2^H_4_‐acetaldehyde and ^2^H_5_‐ethanol as internal standards. A multidimensional approach was used to develop the method, including sample collection and processing, instrumental data analysis, optimization, and validation. Blood and tissues collected from genetically modified mouse models and their wild‐type counterparts were studied.

**Results:**

The method was validated and applied to quantitate acetaldehyde and ethanol in blood and tissues from multiple mouse studies on ethanol metabolism. Acetaldehyde and ethanol were well‐resolved from chromatographic interferences with linear ranges of 6.25–800 μM for acetaldehyde and 1.25–160 mM for ethanol. Both regression coefficients for calibration curves were >0.999. The within‐ and between‐run precisions for ethanol in plasma, whole blood, and serum were all <5.0%, and for acetaldehyde in plasma and serum were <9.0%, while in whole blood it was 19.2%. Sample throughput was on the order of 60 samples per 15 h daily, with a maximum of 111 per batch.

**Conclusions:**

Despite some limitations, this validated method proved to be specific, accurate, and reproducible for high‐throughput quantitation of acetaldehyde and ethanol in rodent plasma, whole blood, serum, and visceral organs.

## Graphical abstract

Applying state‐of‐the‐art technologies and the Direct PCA Procedure, this validated method for quantifying acetaldehyde and ethanol in biological specimens was accurate, reproducible, and high throughput. It has been applied in multiple mouse studies, yielding consistent and sensitive results. Utilizing this analytical method in versatile genetically modified mouse models of alcohol misuse could facilitate important research on ethanol metabolism, supporting the idea that acetaldehyde might play a critical, previously unreported role in ethanol‐related physiologies and pathologies.

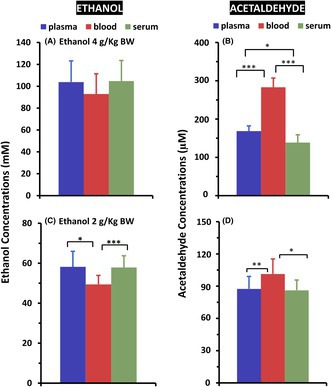

## INTRODUCTION

Misuse of ethanol has been linked to a number of human pathologies, and the underlying mechanisms are still unclear (Avila et al., 2020; Danpanichkul et al., 2025; Gao et al., 2024; Mackowiak et al., 2024). Acetaldehyde, an immediate ethanol metabolite, mediates many ethanol‐induced behavioral effects and is both psychoactive and toxic to animals and humans (Brooks & Zakhari, 2014). Monitoring the kinetics of acetaldehyde using rodent models of ethanol misuse, especially genetically modified mouse models (Isse et al., 2002; Salazar & Centanni, 2024), is essential for understanding and managing ethanol‐associated diseases. However, quantitation of acetaldehyde in biological specimens after alcohol consumption has been challenging for decades since its discovery (Gee & Chaikoff, 1926) due to its high volatility, relatively low concentrations, and strong reactivity toward biochemical molecules (Eriksson, 2001).

Many methods have been developed to determine acetaldehyde concentration using classic techniques (Eriksson, 1973; Klendshoj & Feldstein, 1955; Macchia et al., 1995; Pontes et al., 2009; Truitt, 1970) and modern gas chromatography/ mass spectrometry (GC/MS) in the past two decades (Cordell et al., 2013; Guillot et al., 2019; Heit et al., 2016; Isse et al., 2002; Oh & Park, 2024; Sarkola et al., 2002; Sun et al., 2016). However, these methods yield variable results, likely due to several factors, such as a lack of a suitable internal standard and inconsistent experimental temperatures. For example, propanol and toluene were often used as internal standards (Heit et al., 2016; Isse et al., 2002; McCarver‐May & Durisin, 1997), but their chemical characteristics are drastically different from those of acetaldehyde and are not appropriate as internal standards. In some studies, the experimental temperature was not consistently controlled, which might result in a very low recovery rate (Cordell et al., 2013). As a result, the role of acetaldehyde in ethanol‐related pathologies has not been extensively investigated, posing a challenge to advancing ethanol metabolism research (Enrico & Diana, 2017; Mackus et al., 2020). Thus, it was necessary to develop and establish an accurate and high‐throughput method to quantitate acetaldehyde and ethanol using state‐of‐the‐art technologies. Earlier, we employed stable isotope‐labeled counterparts as internal standards in a modified method, which could measure the analytes of interest accurately (Guillot et al., 2019). However, sample processing was time‐consuming, and the scale of analysis was too small to meet the increasing needs in ethanol‐related studies.

In response to these issues, our laboratory has developed and validated a robust, accurate, and high‐throughput method to quantitate acetaldehyde and ethanol in mouse blood and organs, partially modified from multiple previous procedures (Eriksson et al., 1980; Guillot et al., 2019; Heit et al., 2016; Jin, Cao, et al., 2021). A GC/MS in the positive chemical ionization mode, coupled with a 111‐vial headspace autosampler, was used to determine the concentrations of acetaldehyde and ethanol after samples were treated with diluted perchloric acid containing their stable isotope‐labeled counterparts. Over the years, we have tested and applied this method in several preclinical studies exploring how ethanol metabolism regulates ethanol consumption in rodent models, especially genetically modified mouse models.

## MATERIALS AND METHODS

### Animal studies

The Animal Study Protocol (ASP# LLD‐BG‐1) was reviewed and approved by the Animal Care and Use Committee of the National Institute on Alcohol Abuse and Alcoholism. All animal procedures followed the National Institutes of Health animal care and welfare guidelines. Animals were housed in the NIH animal facility under conventional conditions with a 12‐h light/dark cycle at a room temperature of 22°C and humidity maintained at 55%. After weaning, animals were placed on the NIH Chow diet, and diet and water were provided ad libitum.

Wild‐type (WT) C57BL/6NCr mice were obtained from Charles River (Raleigh, NC) and bred in‐house. Global acetaldehyde dehydrogenase II knockout (*Aldh2* KO) mice (Isse et al., 2002) were initially obtained from Dr. Toshihiro Kawamoto (Japan) and backcrossed to a C57BL/6N background for more than 10 generations and reproduced in‐house at the NIH animal facilities. Adult female animals were studied to exclude possible sex influences observed by multiple groups in clinical studies (Baraona et al., 2001; Erol & Karpyak, 2015) and preclinical alcohol studies (Bizzaro et al., 2023; Eriksson & Sippel, 1977; Jury et al., 2017; Redmond & Cohen, 1972; Salazar & Centanni, 2024).

#### Ethanol gavage and sample collection

Adult animals fasted overnight (5 pm to 9 am) and received a single oral dosage of 2 or 4 g of ethanol per kg of body weight or laboratory tap water as the vehicle control. Ethanol solutions were prepared freshly by diluting the food‐grade ethanol with laboratory tap water (Bertola et al., 2013). Whole blood, plasma, and serum were collected at a single time point of 45 min or a series of time points postethanol gavage. Animals were anesthetized with isoflurane in an induction chamber of a certified Isoflurane Anesthesia vaporizer (Vaporizer Sales & Service Inc., Rockmart, GA) and tested for muscle responses. Blood was collected through the retro‐orbital sinus into prechilled EDTA‐coated tubes (SARSTEDT AG & Co., Numbrecht, GE) for whole blood and plasma and into Eppendorf® tubes without anticoagulant for serum. All sample tubes were capped immediately afterward. Plasma and serum were separated by centrifuging at 1800 *g* for 15 min at 4°C. Whole blood, plasma, and serum were then aliquoted into assay‐ready portions (25–50 μL), frozen over dry ice, and stored at −80°C until further analyses. In the previous study (Fu et al., 2024), the Microvette® 500 Z Gel tube (Sarstedt, Newton, MA) was used to collect serum, and the recommended centrifugation was 10,000 *g* for 4 min at 4°C. After blood collection, mice were euthanized by cervical dislocation and dissected over ice. Small pieces of tissue, about <100 mg each, were frozen in liquid nitrogen or over dry ice and stored at −80°C until analysis. Routinely, samples that were thawed once and refrozen were not applied for acetaldehyde measurement.

### Chemicals

All chemicals were of analytical reagent grade unless otherwise indicated in the text and screened for chromatographic interference by GC/MS analysis. U.S.P. 200 proof anhydrous ethyl alcohol used for administering to animals was obtained from the Warner‐Graham Company (Cockeysville, MD). Acetaldehyde‐^2^H_4_ (^2^H, 99%; chemical purity ≥98%) and ethanol‐^2^H_6_ anhydrous (DLM‐31‐0; ^2^H, 99%; chemical purity ≥99%; a mixture of 2,2,3,3,3‐^2^H_5_‐ethanol & 1,2,2,3,3,3‐^2^H_6_‐ethanol) were purchased from Cambridge Isotope Laboratories (Tewksbury, MA). (Ethanol‐^2^H_5_ was used as the internal standard for ethanol.) Ethanol and acetaldehyde used as analytical standards, perchloric acid (70%), and thiourea were from MilliporeSigma (Milwaukee, WI). HPLC‐grade water submicron filtered (Thermo Fisher Scientific, Waltham, MA) or fresh in‐house distilled water, referred to as water, was used to prepare aqueous solutions but not to administer to animals. Helium at 5.5 Research Grade (99.999%) was purchased from Roberts Oxygen Company, Inc. (Gaithersburg, MD), and methane at Research Purity was purchased from Matheson (Basking Ridge, NJ) through Roberts Oxygen Company, Inc. Chemical wastes were treated according to NIH Chemical Waste Guidelines 2020 and 2023.

The perchloric acid solution of 0.6 N, referred to as PCA0.6 N, was diluted from the purchased perchloric acid using chilled water, aliquoted, and stored at 4°C. Stock solutions of analytical standards were prepared in bulk, aliquoted, and stored at 4°C. The working solution containing internal standards, S0102, was prepared freshly by diluting known amounts of internal standard stock solutions 100‐fold in PCA0.6 N.

Additionally, low‐density polyethylene terephthalate (PET) materials were excluded from the study, and the use of high‐density PET materials was limited. Newly introduced materials, such as vials and tips, were screened for acetaldehyde signals before being applied to the assay.

### Sample processing

The sample processing procedure was referred to as the “Direct PCA Procedure.” In brief, one aliquot of solution S0102 containing PCA0.6 N and internal standards was added to one aliquot of each biospecimen and vortexed, and a portion of the supernatant after centrifugation was transferred for data acquisition in the headspace GC/MS. Perchloric acid, an oxidizing and reducing agent, was applied to denature proteins and disrupt enzyme activities based on previously reported procedures (Eriksson et al., 1977a, 1977b, 1982; Heit et al., 2016; Jin, Cao, et al., 2021) to detach the unstably bound acetaldehyde from proteins and remove protein in the biospecimen. Corresponding calibration curves were established as part of the method validation, as described later in Method Validation, and served similarly as correction curves suggested by Eriksson (Eriksson, 1980) when applying perchloric acid in sample processing. The liquid samples, such as whole blood, plasma, and serum, were treated slightly differently from solid tissues. And the details are described below.

#### Liquid tissues

Aliquots of frozen liquid samples (25–50 μL each) in 1.5‐mL Eppendorf tubes were thawed over ice, and to each was added 250 μL of solution S0102 containing ^2^H_5_‐ethanol (0.5 μmol) and ^2^H_4_‐acetaldehyde (5 nmol), vortexed briefly, then kept on ice. After processing four samples, the plasma or serum was vortexed simultaneously for another 30 s, and a group of whole blood for another 50 s. The mixtures were then centrifuged for 15 min at 13,000 *g* at 4°C, and 200 μL of the supernatant was transferred into a prechilled 20 mL headspace glass vial, followed by immediately crimping the cap to seal. The prepared samples were then loaded onto the headspace platform for data acquisition at room temperature by GC/MS.

#### Solid tissues

An aliquot of frozen solid sample, approximately 15–40 mg in weight, was transferred or split from the quick‐frozen tissues during collection over a glass surface chilled by dry ice and placed into a prechilled 2.0 mL reinforced Precellys® homogenization tubes (Bertin Technologies, France) containing ceramic beads (0.4 mm each), then mixed with chilled 250 μL of solution S0102, capped, and vortexed briefly. The processed samples were kept in the ice while the remaining samples were processed. Next, 24 or fewer samples were homogenized simultaneously at 4°C using a Precellys® Evolution 24‐homogenizer coupled with a dry ice‐filled Cryolys Evolution (Bertin Technologies, France). Samples were homogenized at 5800 rpm for 25 sec twice with an intermittent pause of 20 sec and then centrifuged at 13,000 *g* for 15 min at 4°C. The remaining procedures were identical to those for the liquid samples. Details of sample processing for individual organs and tissues, such as visceral organs and digestive intestines, were presented in previous publications (Fu et al., 2024; Maccioni et al., 2025; Mackowiak et al., 2025).

A preset sequence‐guided data acquisition by GC/MS commenced after the samples were loaded onto the headspace autosampler platform. The complete method for data acquisition by headspace GC/MS is presented in the Data [Link], [Link] and a summary of parameters is presented below.

### Instrumentation

#### Gas chromatography/mass spectrometry, headspace autosampler

An Agilent 8890B GC/5977B MS system coupled with a 111‐vial 7697A headspace autosampler (Agilent Technologies, Inc.; Santa Clara, CA) was employed to quantitate acetaldehyde and ethanol using the stable isotope dilution and selected ion monitoring techniques. Prepared samples stored in 20‐mL headspace glass vials were introduced to the GC/MS through the headspace autosampler. The headspace oven temperature was set at 60°C with a loop temperature of 70°C and a transfer line temperature of 75°C. The vial was loaded and equilibrated in the autosampler, of which 1 mL of each sample in the headspace was injected into the customized GC coupled to the single quadrupole MS.

The GC oven temperature was programmed to increase from 32 to 60°C at a rate of 50°C/min and then continued to 70°C at a rate of 100°C/min with a hold for 1.5 min. This was followed by a postrun of 1.5 min at 220°C. The final oven temperature rested at 100°C after each sequence was finished. The GC inlet was set at 250°C with a split ratio of 200:1 and a constant total flow of 216 mL/min for the carrier gas, helium. A fused‐silica, DB‐Select 624 ultra inert capillary column (Agilent 122‐0334 UI, 30 m × 0.25 mm ID × 1.4 μm film thickness) was used to separate the analytes of interest and introduce them into the MS. The mass spectrometer was operated in positive chemical ionization mode to detect the signal abundances of protonated ions for each analyte using methane as the reaction gas. The MS temperatures were set at 250°C for the MS ion source, 150°C for the quadrupole, and 250°C for the MSD transfer line.

#### Data acquisition

**FIGURE 1:**
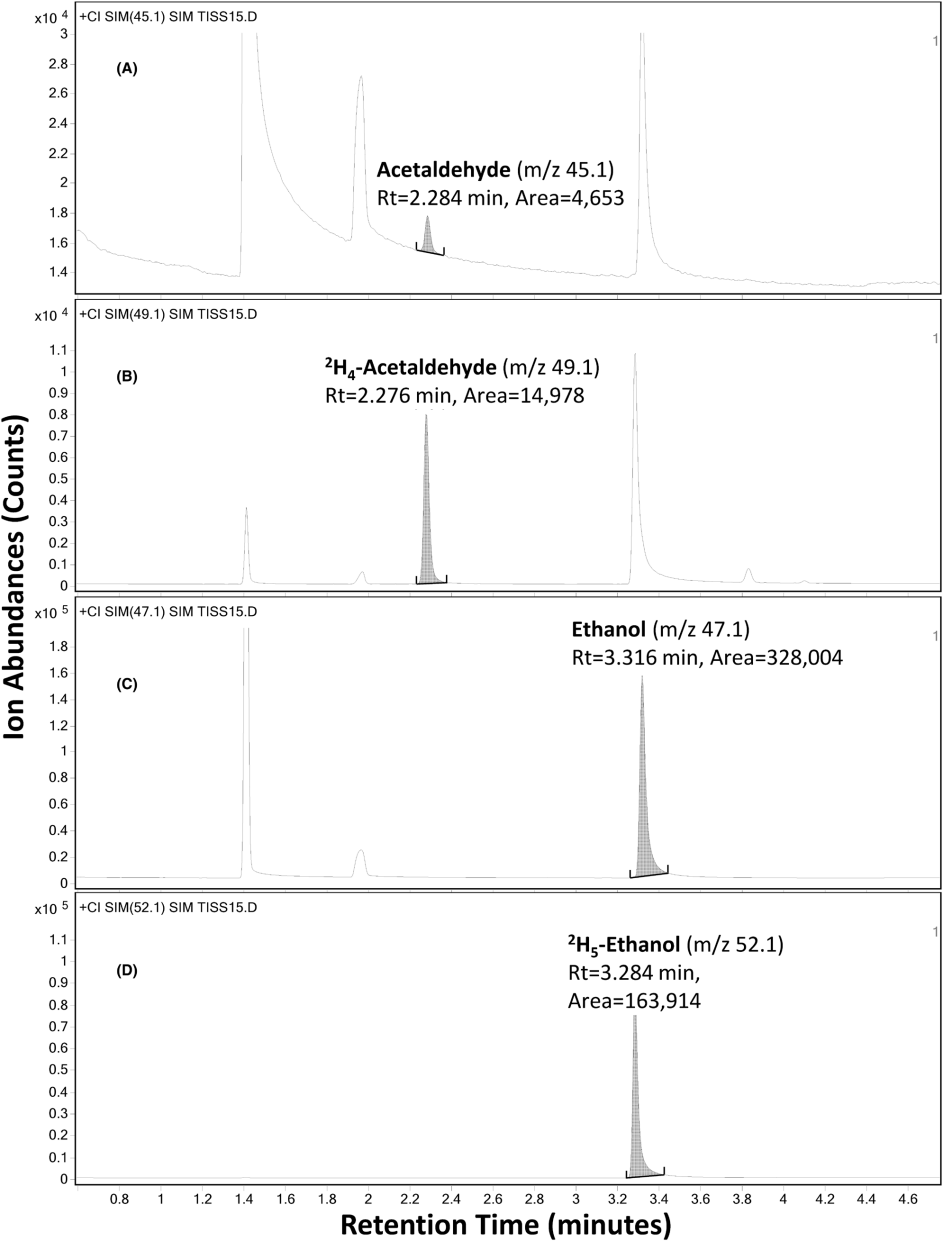
Demonstration of ion chromatograms of mass‐over‐charges for acetaldehyde and ethanol and their internal standards from one mouse serum sample. The ion chromatograms were plotted with the retention time of each ion as the *x*‐axis (minutes) and ion abundance as the *y*‐axis (counts). Details are as follows: (A) *m*/*z* 45.1 for acetaldehyde; (B) *m*/*z* 49.1 for ^2^H_4_‐acetaldehyde; (C) *m*/*z* 47.1 for ethanol; (D) *m*/*z* 52.1 for ^2^H_5_‐ethanol.

Agilent MassHunter Data Acquisition (online), version 10.0, was used to acquire the signal abundances of acetaldehyde and ethanol, along with their internal standards. Each sample run took an average of 15 min, and the daily run‐throughput routinely reached 60 samples per batch in 15 hr, with a maximum capacity of 111 for a single batch. The selected molecular ions (M^+1^) for monitoring were *m*/*z* 45.1 for acetaldehyde, *m*/*z* 49.1 for ^2^H_4_‐acetaldehyde, *m*/*z* 47.1 for ethanol, and *m*/*z* 52.1 for ^2^H_5_‐ethanol. The retention times for each *m*/*z* ion in one mouse serum sample were illustrated in Figure 1 and presented in the Section 3.

### Calculation and data report

The concentration of each analyte was determined by comparing the integrated area under the peak of the individual analyte to that of its internal standard, using the corresponding calibration curve. The responses acquired by GC/MS were analyzed batch by batch using an independent offline computer system. Agilent MassHunter Quantitative Analysis (version 10.2) coupled with Agile 2 Integrator was employed to automatically integrate the signal peaks of the selected ions, to calculate the concentrations of the analytes by batch, and to generate a result report for acetaldehyde and ethanol concentrations in biological samples. Manual integration was applied to correct and integrate the peaks of analytes that were misidentified by the auto‐integrator. Additionally, Excel for Microsoft 365 was used to conduct statistical analysis. A two‐sided, pairwise Student's *t*‐test was applied to determine significant differences between study groups at a *p* value <0.05, 0.01, 0.001, or 0.0001.

### Method validation

The method was validated partially to be consistent with Shah's report (Shah et al., 2000) and the Bioanalytical Method Validation in Guidance for Industry (Docket# FDA‐2013‐D‐1020) by the US Food and Drug Administration (Food and Drug Administration, 2018). In particular, linearity, precision, and recovery were evaluated. The interference from each analyte on the individual ion was also cross‐examined on the chromatogram.

#### Linearity

The linearity of the method was evaluated using a calibration curve for each analyte with mouse plasma as the matrix. The calibration curves were constructed by systematically diluting known concentrations of acetaldehyde and ethanol standard solutions and adding known amounts of internal standards. The series of ethanol concentrations was 1.25, 2.5, 5, 10, 20, 40, 80, 160 mM along with 0.5 μmol of internal standard, while the series of acetaldehyde concentrations was 6.25, 12.5, 25, 50, 100, 200, 400, 800 μM along with 5 nmol of internal standard. The peak area ratio of ion abundance of the analyte to its internal standard was plotted against the ratio of the added amount of analyte to that of its internal standard. An *R* ^2^ > 0.99 was accepted.

#### Recovery

The recovery was assessed at various concentrations of ethanol and acetaldehyde in a blank serum matrix from mice without prior exposure to ethanol. A mixture of nonlabeled acetaldehyde and ethanol standard solutions was prepared at various concentrations and mixed into 25 μL of mouse serum at 4.0, 20.0, 40.0, and 80.0 mM for ethanol, and 40, 200, 400, and 800 μM for acetaldehyde, respectively. The prepared samples were incubated on ice for 15 min and then treated the same as liquid samples, as described in the Method section.

#### Precision, LOQ, and LOD

Precision was expressed as a CV of ethanol and acetaldehyde concentrations in plasma, whole blood, and serum determined from repeated measurements of sample replicates. Plasma, whole blood, or serum was collected at 45 min from *Aldh2* KO mice given 2 g/kg BW of ethanol. Each type of sample was pooled from 2 to 3 mice, mixed, aliquoted in 25 μL each, frozen over dry ice, and stored at −80°C until analysis. The within‐run precision was determined by measuring acetaldehyde and ethanol concentrations in five replicates of the pooled samples. The between‐run precision was determined by repeatedly measuring new sets of five replicates of the pooled samples seven times over about 6 months.

The limit of quantitation (LOQ) was examined in Levels 1 and 2 of ethanol and acetaldehyde concentrations in the corresponding calibration curve in mouse plasma as the matrix. The limit of detection (LOD) was examined in the diluted ethanol and acetaldehyde solutions of Level 1 in the calibration curves in mouse plasma.

### Method applications

This method was applied to compare ethanol and acetaldehyde concentrations in three forms of blood samples: plasma, whole blood, and serum from the same animal. Adult WT or *Aldh2* KO mice were gavaged with a single oral dosage of 2 or 4 g of ethanol per kg of BW, and portions of plasma, whole blood, and serum of each mouse were collected at 0, 2.5, 5, 10, 20, 30, 45, 60 min, 2, 4, and 6 h, or 45 min only post gavage.

Additionally, the method has been applied to determine acetaldehyde and ethanol concentrations in serum, whole blood, liver, brain, and other visceral organs and tissues in multiple studies on ethanol metabolism in our laboratory since 2020.

## RESULTS

### Selected ion chromatograms

Ion chromatograms for acetaldehyde, ^2^H_4_‐acetaldehyde, ethanol, and ^2^H_5_‐ethanol from one mouse serum sample were presented in Figure 1. Under these experimental conditions, the retention time (Rt) for acetaldehyde (*m*/*z* 45.1, Figure 1A) was 2.284 min, for ^2^H_4_‐acetaldehyde (*m*/*z* 49.1, Figure 1B) was 2.276 min, for ethanol (*m*/*z* 47.1, Figure 1C) was 3.316 min, and for ^2^H_5_‐ethanol (*m*/*z* 52.1, Figure 1D) was 3.284 min. Acetaldehyde and ethanol showed good resolution from each other (*R* = 6.55). Each selected ion was specific for each analyte of interest and its deuterium‐labeled internal standard. The baselines for *m*/*z* 52.1 and *m*/*z* 49.1 were <1000 counts (ion abundance), while those for *m*/*z* 47.1 and *m*/*z* 45.1 were approximately 5000 and 13,000 counts, respectively. The latter was quite high. However, chromatographic noise for selected ions of all four compounds was below the limits of detection in the blank control serum, though not for *m*/*z* 45.1 (acetaldehyde) in visceral organs. A signal abundance of *m*/*z* 45.1 at the same Rt as acetaldehyde was observed in control organ samples, but the sources of these signals have not been identified. This is discussed later as part of the method's limitations.

### Method validation (linearity, recovery, precision, LOQ, LOD)

**FIGURE 2:**
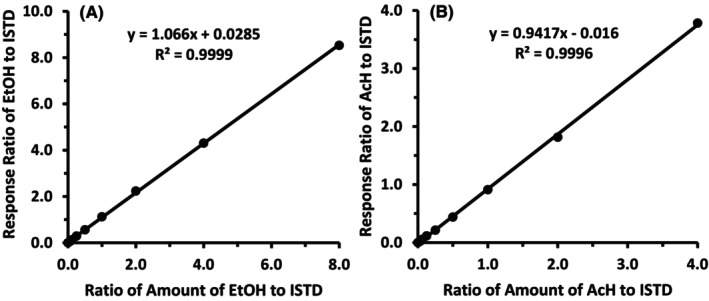
Calibration curves for ethanol and acetaldehyde. The calibration curves were plotted with the ratio of amounts of ethanol and acetaldehyde to respective internal standard (ISTD; *x*‐axis) against the ratio of ion abundances of ethanol and acetaldehyde to their ISTDs (*y*‐axis). The linear regressions were analyzed using Excel for Microsoft 365. (A) EtOH, ethanol; (B) AcH, acetaldehyde.

**FIGURE 3:**
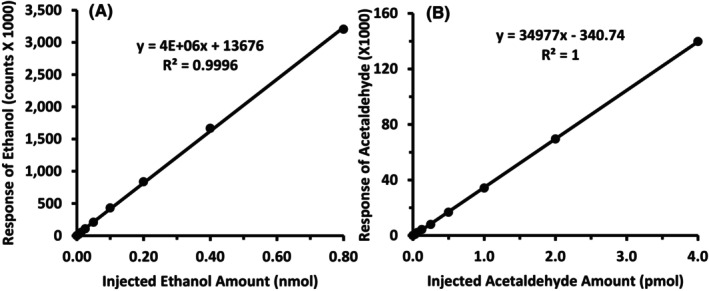
Regression curves for the injected amounts of ethanol and acetaldehyde (*x*‐axis) vs. their responses in GC/MS (*y*‐axis). The linear regressions were analyzed using Excel for Microsoft 365. (A) EtOH, ethanol; (B) AcH, acetaldehyde.

As presented in Figure 2, using the quantification method described for ethanol and acetaldehyde, sample analysis was linear from 1.25 to 160 mM for ethanol and 6.25 to 800 μM for acetaldehyde, with an *R* ^2^ of >0.999 for each. Samples with ethanol concentrations >160 mM were diluted with PBS to fit into the linear range. The amount of ethanol injected versus the responses of ethanol detected by GC/MS showed an *R* ^2^ > 0.999 in the range of 6.25 pmol to 0.80 nmol, and that of acetaldehyde was >0.999 in the range of 31.25 fmol to 2 pmol, as indicated in Figure 3.

The recoveries of ethanol and acetaldehyde were presented in Table 1. All recoveries on average (*n* = 3) were 92% or greater, except 86% for acetaldehyde at 40 μM. Within‐run precisions (*n* = 5) and between‐run precisions (*n* = 7 experiments of 5 replicates) were presented in Table 2, along with the average concentrations of ethanol and acetaldehyde in plasma, whole blood, and serum samples. Precisions (CV) were <5% for ethanol and <9% for acetaldehyde in all samples, except that CV was 19.2% for acetaldehyde in whole blood at an average concentration of 69.0 ± 13.2 μM. The samples described above were pooled from multiple female mice with body weights between 21 and 29 g. We observed that mouse body weight affected the acetaldehyde and ethanol concentrations when mice were gavaged with the same amount of ethanol per kg of body weight (data not presented). Thus, the ethanol and acetaldehyde concentrations in the validation experiments were not compared to those in the method applications below.

**TABLE 1:** The recovery for acetaldehyde and ethanol was examined in WT mouse serum at various concentrations, 4.0, 20.0, 40.0, and 80.0 mM for ethanol and 40, 200, 400, and 800 μM for acetaldehyde.

Recovery for ethanol			Recovery for acetaldehyde		
CONC (mM)	Mean (%) ± SD	CV%	CONC (μM)	Mean (%) ± SD	CV%
4.00	108.0 ± 3.6	3.4	40	86.3 ± 8.9	10.3
20.0	100.3 ± 0.7	0.7	200	98.6 ± 1.8	1.9
40.0	93.9 ± 0.8	0.8	400	95.9 ± 3.7	3.8
80.0	92.1 ± 3.7	4.0	800	93.1 ± 6.0	6.4

*Note*: Data are expressed as mean ± SD (*n* = 3).

**TABLE 2:** Within‐ and between‐run precisions (CV) for acetaldehyde and ethanol were determined in plasma, whole blood, and serum collected from *Aldh*2 KO mice given a single oral dose of 2 g/kg BW of ethanol at 45 min post‐gavage.

Analytes	Plasma		Whole blood		Serum	
	Mean ± SD	CV(%)	Mean ± SD	CV(%)	Mean ± SD	CV (%)
Within‐run precision (*n* = 5 replicates)						
[Ethanol], mM	56.8 ± 1.2	2.1	52.2 ± 1.8	3.5	60.3 ± 1.7	2.8
[Acetaldehyde], μM	96.4 ± 4.0	4.6	66.7 ± 3.8	5.7	98.4 ± 7.5	7.6
Between‐run precision (*n* = 7 experiments of 5 replicates over 6 months)						
[Ethanol], mM	56.5 ± 1.6	2.9	51.5 ± 2.2	4.3	58.5 ± 2.0	3.4
[Acetaldehyde], μM	92.0 ± 8.0	8.9	69.0 ± 13.2	19.2	94.1 ± 7.0	7.5

*Note*: Pooled samples were aliquoted into 25 μL portions and frozen over dry ice upon collection. Within‐run precision was determined (*n* = 5) the next day after sample collection. The between‐run precision was determined (*n* = 7) from seven measurements of five replicates within about 6 months after sample collection. Data are expressed as means ± SD (*n* = 5 or 7).

LOQs were 6.25 μM for acetaldehyde and 1.25 mM for ethanol in the mouse plasma matrix, and LODs were 0.4 μM for acetaldehyde and 0.16 mM for ethanol.

### Method applications

#### Ethanol and acetaldehyde in plasma, whole blood, and serum from the same mouse

##### Appearance and disappearance of ethanol and acetaldehyde in WT mice

**FIGURE 4:**
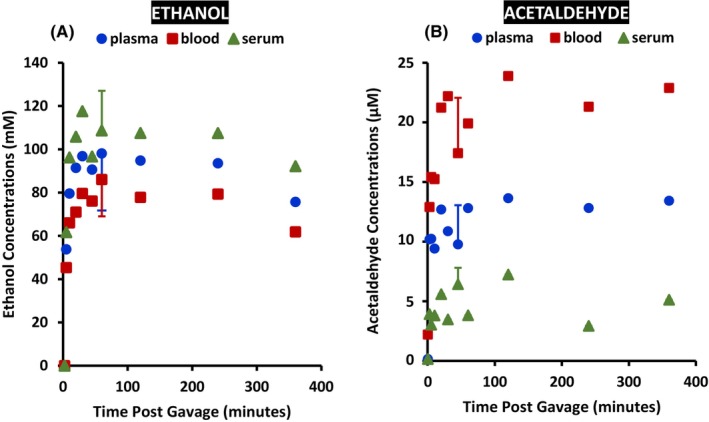
Appearance and disappearance of ethanol and acetaldehyde in WT mouse plasma, whole blood, and serum of the same animal. The ethanol and acetaldehyde concentrations in each component were plotted against the function of time postgavage of a single oral dosage of 4 g/kg BW of ethanol (*n* = 2 animals per time point, except for *n* = 7 at 45 min). (A) ethanol; [Ethanol]_blood@45min_ < [Ethanol]_serum@45min_ at *p* < 0.05. (B) acetaldehyde; [acetaldehyde]_blood@45min_ > [acetaldehyde]_plasma@45min_ at *p* < 0.01 or > [acetaldehyde]_serum@45min_ at *p* < 0.0001; [acetaldehyde]_plasma@45min_ > [acetaldehyde]_serum@45min_ at *p* < 0.05. Statistical analysis was performed via a two‐sided, pairwise Student's *t* test in Microsoft 365 Excel.

The appearance and disappearance of ethanol and acetaldehyde were similar in three forms of blood from each WT mouse over 6 hr. postgavage of a single oral dosage of ethanol at 4 g/kg BW (*n* = 2 mice per time point), as presented in Figure 4. The ethanol concentrations in plasma, whole blood, and serum appeared to be the highest around 30 min postgavage and decreased gradually over 6 h. The acetaldehyde concentrations in all three forms reached a maximum value at a similar time, which was slightly later than those of ethanol, and maintained plateaus of up to 6 h. in plasma and blood. Though the peak time was similar for each component, the concentrations of ethanol and acetaldehyde were different. Among them, serum presented the highest ethanol concentration, followed by plasma and whole blood. In contrast, serum showed the lowest acetaldehyde concentration, and whole blood presented the highest at each time point over 6 hr. This phenomenon was consistent in samples collected at each time point from mice given one oral dose of ethanol.

##### Ethanol and acetaldehyde in WT mice at a single time point of 45 min

Similar patterns were observed in different forms of blood samples when the animal number increased to *n* = 7 at the 45‐min time point under the same conditions as in the above study. As presented in Figure 4A, ethanol concentrations at 45 min (mean ± SD, *n* = 7) were 108.7 ± 18.3 mM in serum, 98.1 ± 26.3 mM in plasma, and 86.0 ± 17.0 mM in blood. The ethanol concentrations in blood were lower in plasma (n.s.) and significantly lower than those in serum (*p* < 0.05). The acetaldehyde concentrations (Figure 4B) were 17.4 ± 4.6 μM in blood, 9.7 ± 3.3 μM in plasma, and 6.4 ± 1.4 μM in serum. The blood acetaldehyde concentrations were significantly greater than those in plasma (*p* < 0.01) and serum (*p* < 0.0001), while plasma acetaldehyde concentrations were greater than those in serum (*p* < 0.05).

##### Ethanol and acetaldehyde in Aldh2 KO mice at a single time point of 45 min

**FIGURE 5:**
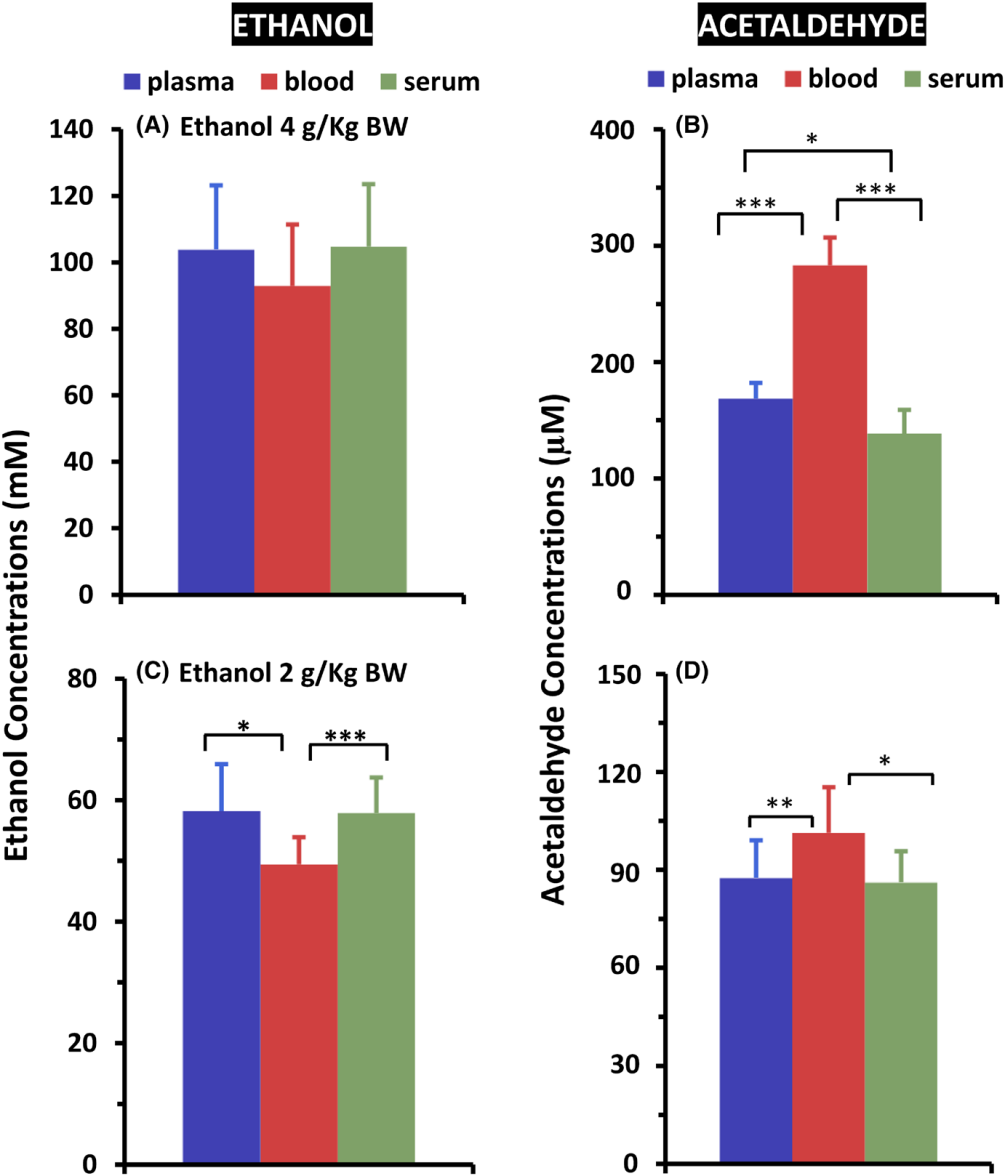
One‐time point comparisons of ethanol and acetaldehyde concentrations in *Aldh2* KO mouse plasma, whole blood, and serum of the same animal. Samples were collected 45 min after mice were given a single oral dosage of ethanol (*n* = 5 each group) at 4 g/kg BW, (A) ethanol and (B) acetaldehyde, and at 2 g/kg BW, (C) ethanol and (D) acetaldehyde. Statistical analysis was performed via a two‐sided, pairwise Student's *t*‐test in Microsoft 365 Excel with indicated *p* values of * < 0.05, ** < 0.01, and *** < 0.001.

Similar tendencies were also observed in the three forms of blood samples from *Aldh2* KO mice given a single oral gavage of ethanol at 2 or 4 g/kg BW. The ethanol concentrations (mean ± SD, *n* = 5) in plasma, whole blood, and serum in *Aldh2* KO mice given 4 g/kG BW were 103.8 ± 19.4, 92.9 ± 18.5, and 104.8 ± 18.7 mM (Figure 5A), and acetaldehyde concentrations were 168.6 ± 13.6, 283.0 ± 24.1, and 138.6 ± 20.3 μM (Figure 5B), respectively. When the mice were given a lower dose, 2 g/kg BW, both the ethanol and acetaldehyde concentrations in all samples decreased but presented similar patterns to those in the 4 g/kg BW group, that is, the whole blood sample had the lowest ethanol concentration but the highest acetaldehyde concentration among the three components from the same animal. Ethanol concentrations (mean ± SD, *n* = 5) were 58.2 ± 7.8, 49.4 ± 4.5, and 57.9 ± 5.8 mM (Figure 5C), and acetaldehyde concentrations were 87.5 ± 11.6, 101.4 ± 14.0, and 86.1 ± 9.6 μM (Figure 5D) in plasma, whole blood, and serum, respectively.

#### Mouse ethanol metabolism studies

The applications of this method in ongoing ethanol metabolism studies in our laboratory were presented briefly in Table 3. In general, genetically modified mouse models and their WT counterparts were administered ethanol or acetaldehyde orally or intraperitoneally at varied dosages. The ethanol and acetaldehyde concentrations were quantitated in blood samples and organs from mice given ethanol or acetaldehyde at various time points. The details of the sample processing for individual organs and results were reported in the published studies, respectively (Fu et al., 2024; Jin, Cao, et al., 2021; Jin, Cinar, et al., 2021; Maccioni et al., 2025; Mackowiak et al., 2022, 2025; Park et al., 2023; Ren et al., 2020).

**TABLE 3:** A brief overview of the applications of this method in ongoing projects related to ethanol metabolism in our laboratory since 2020.

Mouse	Sex	Ethanol administration	Tissues analyzed	References (first author, year)
C57BL/6J	Male	Oral gavage, 5 g/kg BW	Serum, liver	Ren et al. (2020)
C57BL/6N & Genetically modified mice	Male	Intraperitoneally, 1, 2, & 3.6 g/kg BW	Blood, cerebellum	Jin, Cao, et al. (2021)
C57BL/6J & Genetically modified mice	Male	Intraperitoneally, 2 g/kg BW	Serum, spinal cord	Jin, Cinar, et al. (2021)
C57BL/6N & Genetically modified mice	Male	Oral gavage, 5 g/kg BW	Blood	Mackowiak et al. (2022)
C57BL/6N	Male	Intraperitoneally, ethanol 6 g/kg BW; acetaldehyde 50 mg/kg BW	Serum, liver, white adipose tissue; serum	Park et al. (2023)
C57BL/6N & Genetically modified mice	Male & female	Oral gavage, 5 g/kg BW; Intraperitoneally, ethanol 4 g/kg BW; Intra‐intestinal injection of acetaldehyde; Liver perfusion with ethanol	Serum, liver, brain regions, bile fluid, and duodenal luminal content	Fu et al. (2024)
C57BL/6N & Genetically modified mice	Male & female	Oral gavage, 5 g/kg BW; Drinking‐in‐dark, 20% ethanol; Two‐bottle choice, 3% ‐ 20% ethanol;	Whole blood, liver, and brain	Mackowiak et al. (2025)
C57BL/6 & Genetically modified mice	Male & female	Chronic‐plus‐single binge, chronic‐plus‐multiple binges, multiple binges of ethanol	Serum, blood, duodenal content	Maccioni et al. (2025)

## DISCUSSION

Compared to reported methods (Table 4), the current method demonstrated specificity, reproducibility, and accuracy with a high‐throughput capacity for determining acetaldehyde and ethanol in rodent blood and organs, and may be applicable to clinical studies. The data from these application studies have been consistent, reproducible, and sensitive. Utilizing this analytical method in multiple genetically modified mouse models of alcohol misuse has facilitated important research on ethanol metabolism, supporting the idea that acetaldehyde might play a critical, previously unreported role in ethanol‐related physiologies and pathologies.

**TABLE 4:** Comparisons of analytical methods determining acetaldehyde and ethanol in rodents and human biospecimens.

Instrument	[AcH], μM	[EtOH], mM	Tissues (type, amount)	Subjects	ISTD	Sample prep	Misc info	References (first author, year)
Colorimetry	40–400	n/a	Blood, 3 mL	n/a	n/a	Colorimetric reactions		Klendshoj and Feldstein (1955)
Immunosorbent			Human liquid cell				Hemoglobin‐AcH adducts	Niemela and Israel (1992)
Immunologic	adducts		Human					Sillanaukee et al. (1992)
GC/FID HS	16–44 117–217 nmol/g	30 27–30 μmol/g	Blood 200 μL Liver 1.5 g	Rat, EtOH, ip 1.5 g/kg BW	t‐Butyl alcohol & external standard	PCA0.6 N, thiourea	Pentobarbital influence	Eriksson (1973), Eriksson et al. (1977a), Truitt (1970)
GC/FID HS	20 (52) 2–5 (10–13)	15–17 20–22	Blood Urine	Human				Tsukamoto et al. (1989)
GC/FID HS	n/a	0.01–20 g/L	Blood, serum, plasma, urine, saliva, 100 μL		n/a	Not treated		Macchia et al. (1995)
GC/FID HS, Carbowax	0.22–114	2.2–33	Blood, 1 mL	Human	1‐Propanol	PCA0.6 N, inhibitor	No centrifugation	McCarver‐May and Durisin (1997)
GC/FID HS	0.3–3.5	2–12	Blood, 0.5 mL	Human	n/a	PCA0.6 N, 4°C	Nonenzymatic conversion	Eriksson et al. (1982), Sarkola et al. (2002)
GC/FID HS	n/a	80	Blood, 20 μL	Mouse	n‐Propanol		Time‐course study	Livy et al. (2003)
GC/FID HS, CPWAX 57 CB	0.17–5.5 mM	1.6–52	Human	In vitro	1‐Propanol	TritonX‐100, acetonitrile	Validated	Pontes et al. (2009)
HPLC	0.4–6.5	n/a	Plasma RBC, 1 mL	Human	External standard	Derivatized		Peterson and Polizzi (1987)
HPLC	3–80	n/a	Blood, plasma, Cell media: 70 μL	Rat	External standard	Derivatized	Stable	Guan et al. (2012)
GC/MS, stainless steel column	n/a	n/a	In vitro	Human	n/a	Room temperature	Qualitative	Truitt (1970)
GC/MS EI, HS DBWAX			Blood 50–200 μL Tissue 200 mg	mice, two bottles	n/a			Eriksson et al. (1982), Isse et al. (2002)
GC/MS EI, CC‐PAL‐HS, DBWAX	2.3–2273		Plasma 100 μL (leftover)	Human	n‐propanol	No sample processing	Validated, recovery 53%	Cordell et al. (2013)
GC/MS EI, HS Inertcap AQUATIC	2–10 μM 2.5‐15 nmol/g 1–7 nmol/g	4–20 mM 4–15 μmol/g 8–42 μmol/g	Blood <0.5 mL Liver Brain	Mouse, 2 g/kg, ip	toluene	PCA0.6 N, thiourea	Validated	Heit et al. (2016)
GC/MS EI, HS	30 14	100 60 μmol/g	Plasma 100 μL Liver 100 mg	Mouse	n‐propanol	NaCl & water		Sun et al. (2016)
GC/MS EI	10–1000	1–1000	Serum, 50 μL	Mouse	EtOH‐^2^H_2_ AcH‐^2^H_4_	TritonX‐100, acetonitrile	Low throughput	Guillot et al. (2019), Pontes et al. (2009)
GC/MS/MS, HS	0.2–20 μg/mL	20–2000 μg/mL	Plasma 200 μL	Human	Tert‐butanol	NaFl, thiourea, and water	Validated	Oh and Park (2024)
GC/MS PCI, HS	6.25–800	1.25–160	Blood 25 μLSolid tissue 25 mg	Mouse	EtOH‐^2^H_6_ AcH‐^2^H_4_	Direct PCA Procedure	Validated High throughput	Lin et al. (in‐print)

*Note*: Analytical instrument, internal standard, sample preparation, and other relevant information were summarized briefly.

Abbreviations: AcH, acetaldehyde; Blood, whole blood; EI, electron impact; EtOH, ethanol; FID, flame ionization detector; GC, gas chromatograph; HS, headspace autosampler; ISTD, internal standard; MS, mass spectrometer; n/a, data not available; PCA, perchloric acid (0.6 N); PCI, positive chemical ionization.

Key factors in developing this method included the selection of stable isotope‐labeled internal standards, mass‐selected detection for all analytes, the selection of a GC capillary column, and procedural optimization. The internal standard for ethanol, a mixture of andryous ^2^H_5_‐ethanol and ^2^H_6_‐ethanol, was selected from multiple commercially available stable isotope‐labeled ethanols at the time for its highest chemical purity (≥99%), highest isotope purity (D, 99%), and the lowest baseline noise with *m*/*z* 52.1 ion channel. Any commercially available stable isotope‐labeled ethanol, such as pure ^2^H_5_‐ethanol, could serve as an internal standard for ethanol as long as it meets purity standards.

Various polarities of GC capillary columns were examined to optimize the resolutions and sensitivities of analytes and to minimize column bleed. Three columns, HP‐5MS UI (Agilent 19091S‐433UI‐Key; low polarity; 30 m × 0.250 mm ID × 0.25 μm film thickness), DB‐Select 624 UI (medium polarity), and DB‐WAX UI (Agilent 122‐7032UI; high polarity; 30 m × 0.250 mm ID × 0.25 μm), were examined. The medium polarity column 624 UI was selected for its optimal signal resolution between ethanol and acetaldehyde, as well as for the lowest column bleed that contributed to overall baseline noise. The factors influencing the chromatographic baseline for acetaldehyde are discussed below, along with the limitations of this method.

### Interferences of acetaldehyde

Two main sources contributing to the rise of background noise at *m*/*z* 45.1 (acetaldehyde) in the ion chromatogram were identified and minimized or mitigated during sample collection, processing, and data acquisition. One was the GC column bleed, and the other was the plastic consumables used in the experiment.

#### GC column bleed and instrumental operation temperature

One main source interfering with the acetaldehyde signal was unidentified compounds released from the stationary phase components of the GC capillary column, especially at a GC oven temperature of 80°C or higher. This column bleed caused additional irregular baselines for *m*/*z* 45.1 (acetaldehyde) in the ion chromatogram. In addition to the GC oven temperature, the operating temperatures across the system, including headspace autosampler, also contributed to the rise of the baseline for the *m*/*z* 45.1 ion channel.

To minimize the baseline noise for *m*/*z* 45.1, GC and headspace operating temperatures were lowered from those optimized for peak shapes and signal abundances of the analytes. For example, in the applied method, the initial GC programmed oven temperature was 32°C, which was decreased from the optimized 50°C, while the final oven temperature was 70°C, decreased from the optimized 220°C. The temperatures for headspace oven/loop/transfer line were 60/70/75°C in the current method, which were lowered from the previously optimized 80/90/95°C. However, lowering the system temperature caused it to build up moisture and decrease sensitivity (data not presented). Routinely baking the system at high temperatures, replacing gas filters on schedule, and maintaining the resting GC oven temperature at 100°C were necessary to minimize moisture and keep the instrument in good condition.

#### Plastic consumables emission

The other source of interference was introduced by PET‐made laboratory consumables. We observed that the low‐density PET‐made materials, such as containers, persistently emitted small or large amounts of acetaldehyde into the solutions. This caused a significant amount of acetaldehyde in blank control samples. For example, distilled water stored in such containers presented acetaldehyde at various concentrations, depending on the duration of the water stored in the containers (data not presented). This observation agreed with the reports in the food industry (Mutsuga et al., 2006; Re Depaolini et al., 2020) that acetaldehyde and other aldehydes were detected in drinking water in PET bottles, and the acetaldehyde level has been a critical food safety index for bottled drinking water. The high‐density PET‐made laboratory consumables emitted an undetectable amount of acetaldehyde compared to the low‐density PET ones. However, we observed that acetaldehyde in high‐density PET consumables could accumulate over time. Therefore, refraining from using plastic materials during sample preparation is necessary.

Several approaches were applied to reduce the use of plastic materials in acetaldehyde measurement. The low‐density PET materials were eliminated from the method and replaced with glassware, Teflon‐lined, or Teflon‐made materials. The high‐density PET materials were restrained to those of necessity, such as quantitative tips for micro volumes, Eppendorf® tubes, and homogenization tubes. Any newly introduced materials were screened for background noise before being used in the experiment. If possible, leave the materials open to the air or heat them to remove the acetaldehyde, as Gee and Chaikoff reported (Gee & Chaikoff, 1926) that the acetaldehyde observed in the filter paper was removed by heating. Therefore, examining these interferences at the beginning of determining acetaldehyde is necessary for the accuracy of the results.

### Nonspecific enzymatic conversion

Nonspecific enzymatic production of acetaldehyde from ethanol was observed in our studies when samples were processed at room temperature (data not presented). However, controlling experimental temperatures at 4°C or lower, such as quickly freezing samples on dry ice or in liquid nitrogen, storing them at −80°C, and processing them during chemical analysis at 4°C or lower minimized this artifact to between below the limit of detection and 5 μM when ethanol (160 mM) was added to the plasma matrix (*n* = 5). However, Eriksson et al. (Eriksson et al., 1977a) reported that whether blood was frozen quickly or not had no impact on its acetaldehyde concentration. In our study, in addition to controlling experimental temperature, the nonspecific conversion could also be monitored by applying the deuterated ethanol in the blank control sample before the sample was precipitated with PCA0.6 N. Then, the control sample was treated the same as the rest of the study samples, and the occurrence of the signal of ^2^H_4_‐acetaldehyde converted from ^2^H_5_‐ethanol was monitored in GC/MS chromatograms. Any significant peak for the ^2^H_4_‐acetaldehyde that appeared in the above control sample indicated that artifact conversion occurred, and it was necessary to check whether the experimental procedure was controlled correctly. Furthermore, the calibration curves for ethanol and acetaldehyde in our method were constructed simultaneously in the plasma matrix, which could serve a function similar to the correction curve reported in an earlier article (Eriksson, 1980) to correct possible minor nonspecific conversion of acetaldehyde from ethanol.

On the other hand, we also examined the effect of thiourea on nonspecific enzymatic conversion. Thiourea was commonly used to prevent the nonspecific enzymatic conversion of acetaldehyde from ethanol in previous reports (Eriksson et al., 1977a, 1980; Eriksson & Sippel, 1977; Heit et al., 2016). To examine this factor, we compared the effect of thiourea on acetaldehyde concentrations in samples from *Aldh*2 KO mice given ethanol at 2 g/kg BW. The plasma acetaldehyde and ethanol concentrations showed no significant differences between the samples added thiourea (20 mM) and those not, either at the time of sample collection or immediately before chemical analysis after being stored at −80°C (the data were presented in Table S3). The acetaldehyde levels were not increased in samples without the addition of thiourea, and thus, artifactual acetaldehyde converted from ethanol was not observed in these samples. Therefore, this method might minimize the nonspecific enzymatic conversion of acetaldehyde from ethanol without the use of thiourea. Additionally, thiourea is considered carcinogenic (NTP (National Toxicology Program), 2021). Considering the laboratory safety and the minimal nonspecific enzymatic conversion observed in our samples, we decided not to apply thiourea in the current method. However, monitoring artifactual acetaldehyde is recommended when applying this method initially and subsequently. Adding thiourea (20 mM) during sample processing is recommended if the artifactual acetaldehyde persists in the applications of this method.

### Whole blood, plasma, and serum

Whole blood, plasma, and serum are the most accessible biospecimens in animal studies and clinical trials. Our results indicated that any of the three biospecimens could be applied to determine acetaldehyde concentrations in the circulation with some variation. In mice given either 2 or 4 g of ethanol per kg BW, acetaldehyde concentrations were consistently the highest in whole blood, followed by plasma and serum in both WT and *Aldh2* KO mice. Based on the percentage of plasma volume in the whole blood of female mice, which accounts for 65.3% (Riches et al., 1973), the amount of acetaldehyde in the plasma portion of 25 μL of whole blood at 45 min postgavage was approximately accounted for as 40% in both WT and *Aldh*2 KO mice given 4 g/kg ethanol. This indicated that erythrocytes carried greater acetaldehyde than plasma. This finding partially agreed with previous studies in which plasma acetaldehyde concentrations were a fraction of those in whole blood in rats (Eriksson et al., 1977b) or erythrocytes in human subjects (Baraona et al., 1987), although different chemical analysis methods were used. These results also indicated that acetaldehyde might be abundantly bound to proteins in erythrocytes to form adducts in the whole blood, which included mainly hemoglobin (McCarver‐May & Durisin, 1997; Sillanaukee et al., 1992; Tominaga et al., 2009; Westermeyer, 1992; Zakhari, 2006) and some other proteins, such as membrane proteins and soluble proteins (Niemelä, 2007). In clinical studies on alcohol misuse patients, erythrocyte acetaldehyde concentrations in red blood cells were greater in patients with alcohol misuse than in normal subjects (Baraona et al., 1987), and acetaldehyde‐hemoglobin adducts could serve as a marker of alcohol misuse (Niemela & Israel, 1992). Further studies on the variation of acetaldehyde concentration in various forms of blood samples may be important for understanding how acetaldehyde is transported in the circulatory system and among different organs and tissues in preclinical and clinical studies related to ethanol metabolism.

However, analyzing acetaldehyde in whole blood needed extra attention compared to plasma and serum. Coagulated blood was not suitable for acetaldehyde assay, and acetaldehyde concentrations in whole blood showed a greater coefficient of variation than those of plasma and serum (Table 2). Homogenizing whole blood similarly to visceral organs could be a good alternative to minimize the variation of acetaldehyde measurement in whole blood (data not presented).

### Quality control

Quality control at each procedure was necessary to achieve accurate results, including but not limited to training researchers professionally, maintaining consistent internal standard concentrations, collecting and preparing samples at 4°C or lower, maintaining the instrument at optimal conditions, and monitoring intralaboratory results. The intralaboratory reference samples could be prepared from *Aldh2* KO mice (22–25 g BW) and collected at 45 min postgavage of ethanol at 2 g/kg BW, or as appropriate for the corresponding studies. It was also necessary to aliquot and store internal standard stock solutions in small portions (~1 mL) in small amber glass vials, as well as to prepare the working solution freshly.

### Limitations

There are several constraints of the current method. First, the selected GC capillary column was not optimal for the measurement, though it yielded the least column bleed and best resolution of analytes among the three different polarity columns. The chromatographic baseline for *m*/*z* 45.1 was relatively high than those of other ions monitored. Additionally, the peaks of *m*/*z* 47.1 and *m*/*z* 52.1 appeared asymmetric and tailed. These may cause integration bias in low‐abundance signals and among different researchers. Further improvement in column performance is needed. A possible solution could be to collaborate with the manufacturer to customize capillary columns for better compatibility with the acetaldehyde and ethanol assay.

Second, the instrument sensitivity decreased in our older headspace GC/MS system in recent years for unknown reasons. The system occasionally failed to achieve the required methane flow rate of 20%, which probably lowered the instrument sensitivity. A combination of the moisture built up and the presence of perchloric acid in the prepared samples might contribute partially to it. However, the perchloric acid of 0.6 N solution has been widely applied to precipitate protein in ethanol and acetaldehyde measurement (Eriksson et al., 1982; Heit et al., 2016), and we have not found a report on its effect on instrument performance, nor have we observed an immediate or a direct effect over the years. Timely preventive maintenance by professionals and routine maintenance in the laboratory helped keep the instrument functioning as well as possible.

The next issue was that the instrumental integrator, Agile 2, exhibited inconsistency in automatically integrating peaks below LOQ. Monitoring integration accuracy and manually integrating peaks were necessary to ensure accurate results. These obstacles increased personal biases, slowed down reporting results, reduced assay turnover rates, and increased the labor costs. Further improvements are needed in integrating instrumental data.

The other issue was that the signal abundance of *m*/*z* 45.1 at the same Rt as acetaldehyde in the ion chromatogram was detected more or less in most solid tissues, such as visceral organs, feces, and urine of mice without being administered ethanol. This background noise of acetaldehyde interfered with that in samples from mice given ethanol. The current solution to this issue was to monitor the acetaldehyde signal in nonethanol control samples in parallel with the study samples, and any background acetaldehyde was subtracted if it occurred. Only the results calculated from the signal‐to‐noise ratio greater than three in the study samples were accepted. Confirming whether the noise is acetaldehyde or not is also meaningful for ethanol metabolism studies.

Finally, the cost of using this method was relatively high if the sample flow was low. In addition to the initial investment in personnel, laboratory space, and instruments, costs associated with operations included, but were not limited to, instrument preventive maintenance by professionals, wear and tear on the instruments, staff training, and consumables.

## SUMMARY

With the limitations stated, this validated headspace GC/MS‐positive chemical ionization analysis proved that the quantitation of acetaldehyde and ethanol in rodent specimens was highly specific, accurate, reproducible, and high–throughput.

## FUNDING INFORMATION

Disclaimer: This research was supported by the Intramural Research Program of the National Institutes of Health (NIH). The contributions of the NIH authors were made as part of their official duties as NIH federal employees, are in compliance with agency policy requirements, and are considered Works of the United States Government. However, the findings and conclusions presented in this paper are those of the authors and do not necessarily reflect the views of the NIH or the U.S. Department of Health and Human Services.

## CONFLICT OF INTEREST STATEMENT

The authors declare no conflicts of interest related to this study.

## Supporting information


Figure S1

Figure S2

Figure S3

Figure S4

Figure S5

Tables S1–S3

Data S1

Data S2

## Data Availability

The data that support the findings of this study are available from the corresponding author upon reasonable request.
