# Supplementary figures and images for: High‐throughput quantitation of acetaldehyde and ethanol in mice using gas chromatography/mass spectrometry positive chemical ionization
Source: Alcohol Clin Exp Res (Hoboken). 2025 Aug 4;49(9):1897–911. doi: 10.1111/acer.70126 (PMC12463767; doi:10.1111/acer.70126)

**Figure 1.**

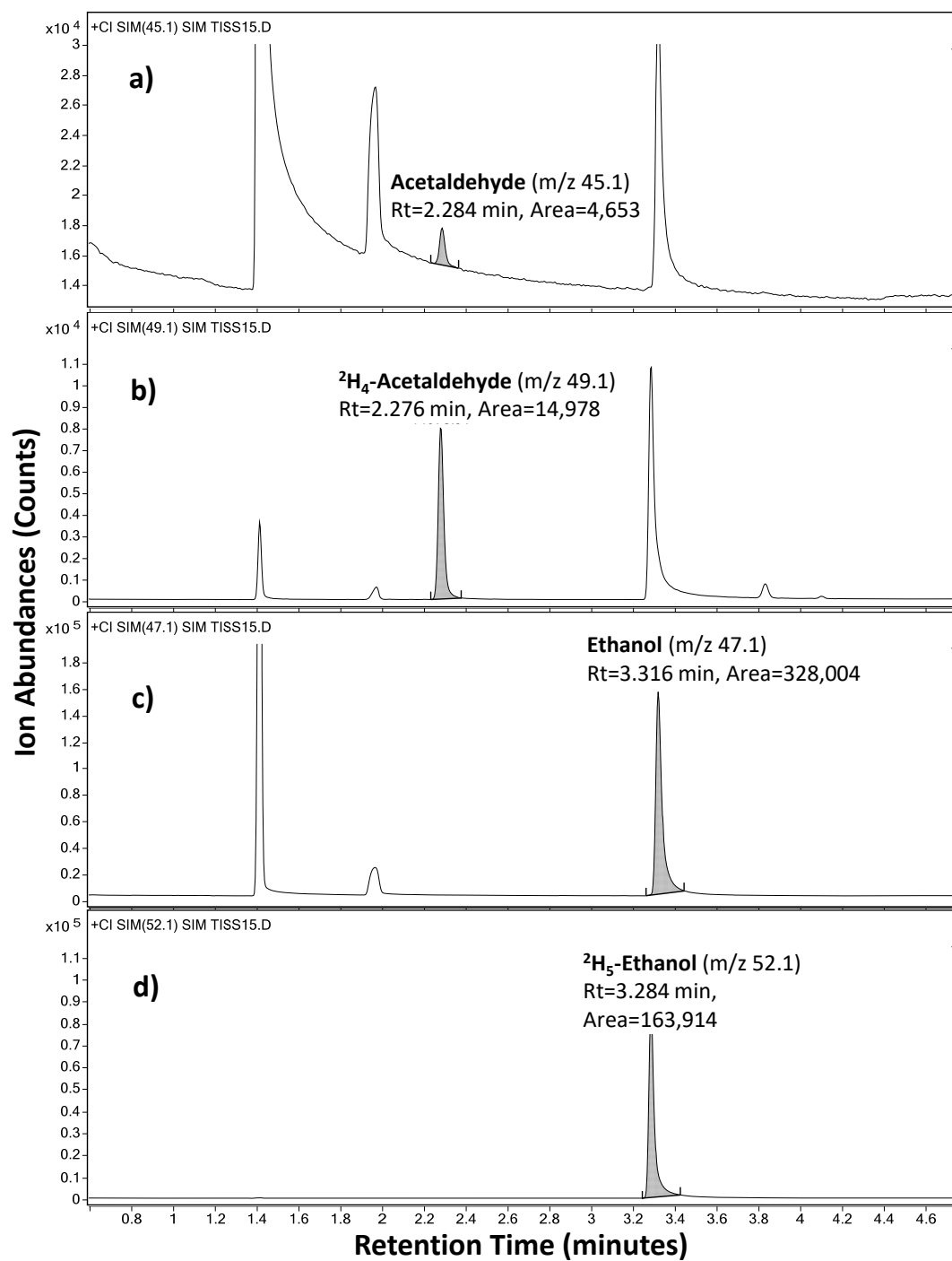

Supplement: Supplementary file 1 — Figure S1 [file ACER-49-1897-s001.pdf]

Figure 2.

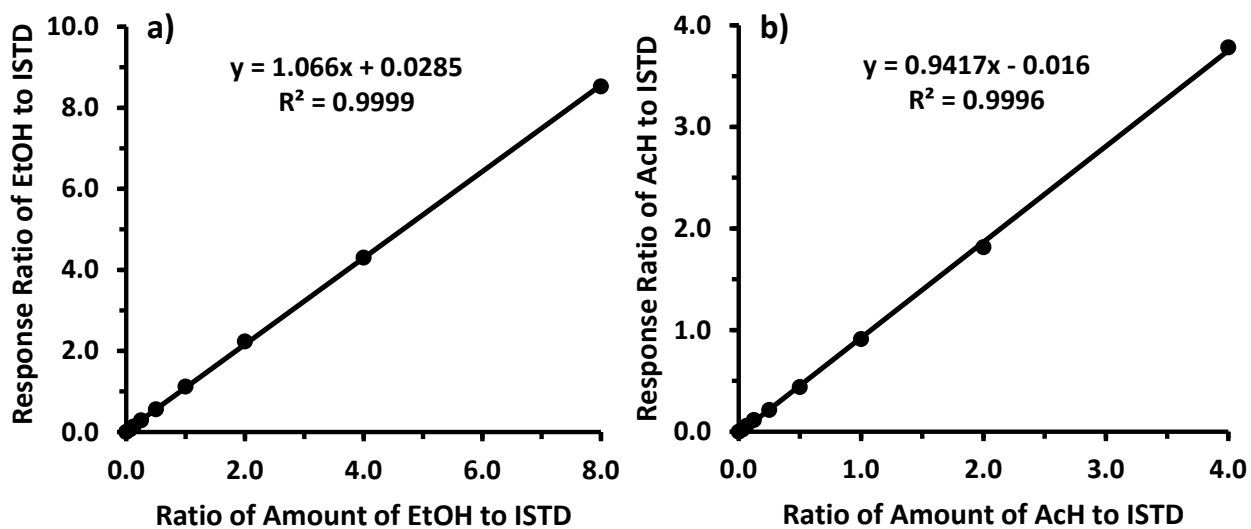

Supplement: Supplementary file 2 — Figure S2 [file ACER-49-1897-s007.pdf]

**Figure 3.**

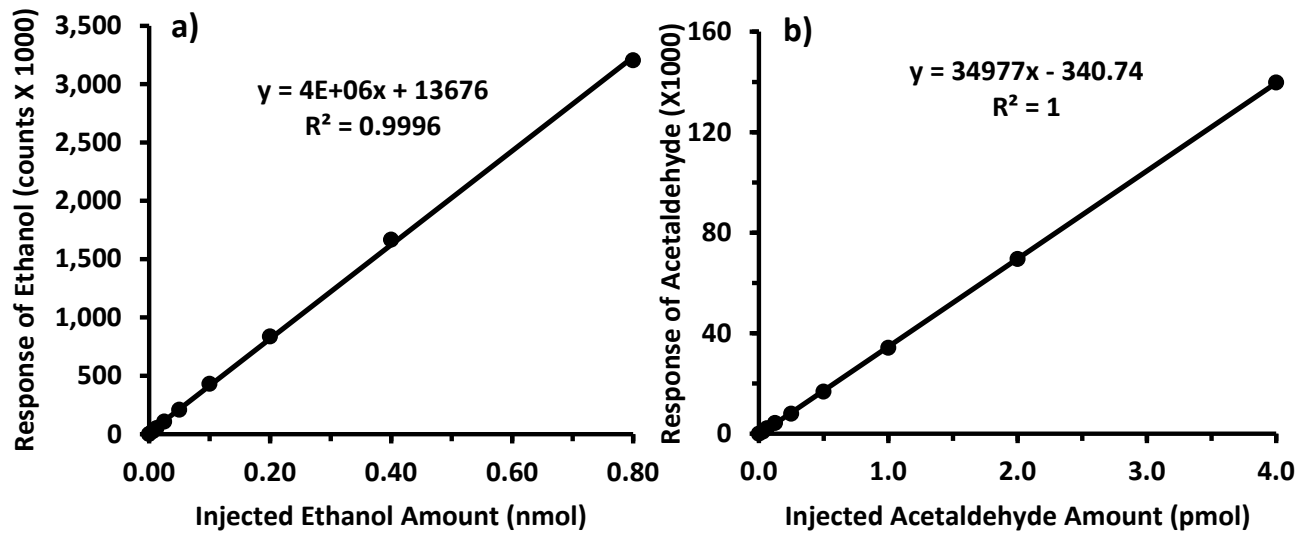

Supplement: Supplementary file 3 — Figure S3 [file ACER-49-1897-s004.pdf]

Figure 4.

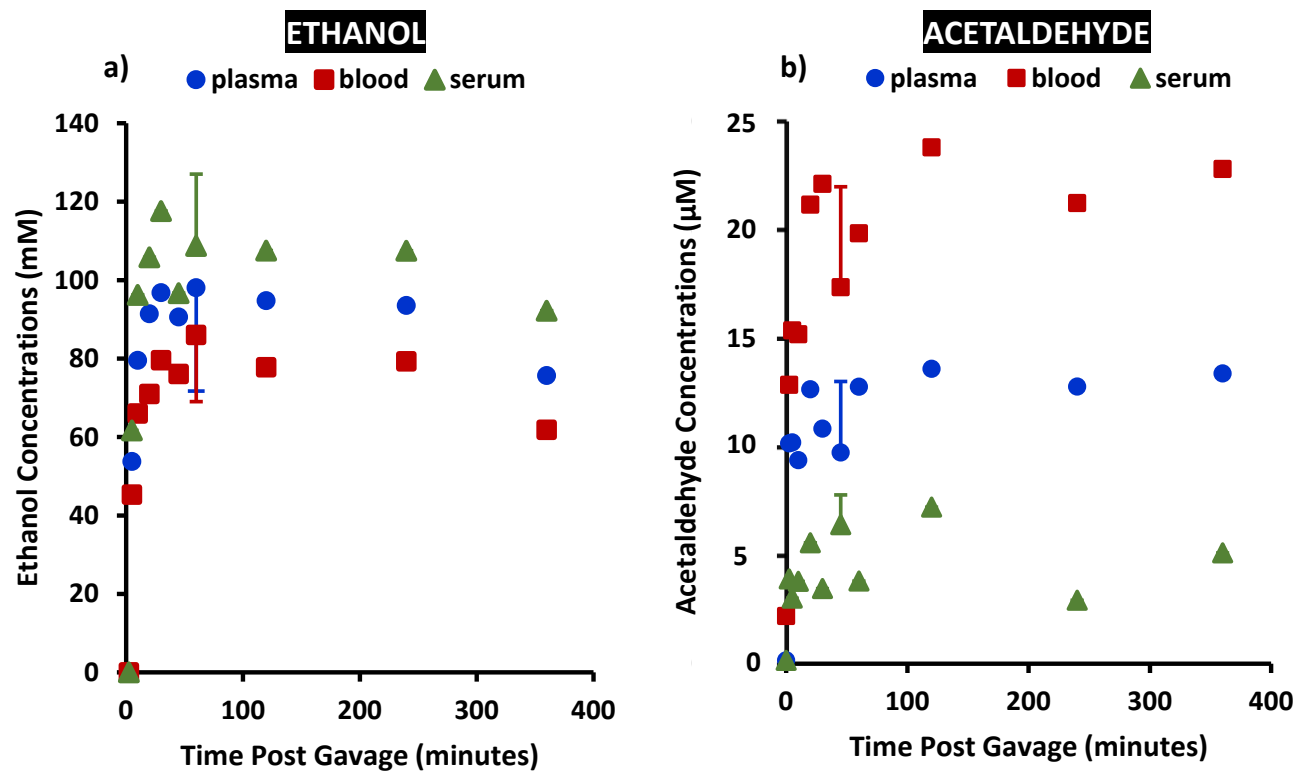

Supplement: Supplementary file 4 — Figure S4 [file ACER-49-1897-s002.pdf]

Figure 5.

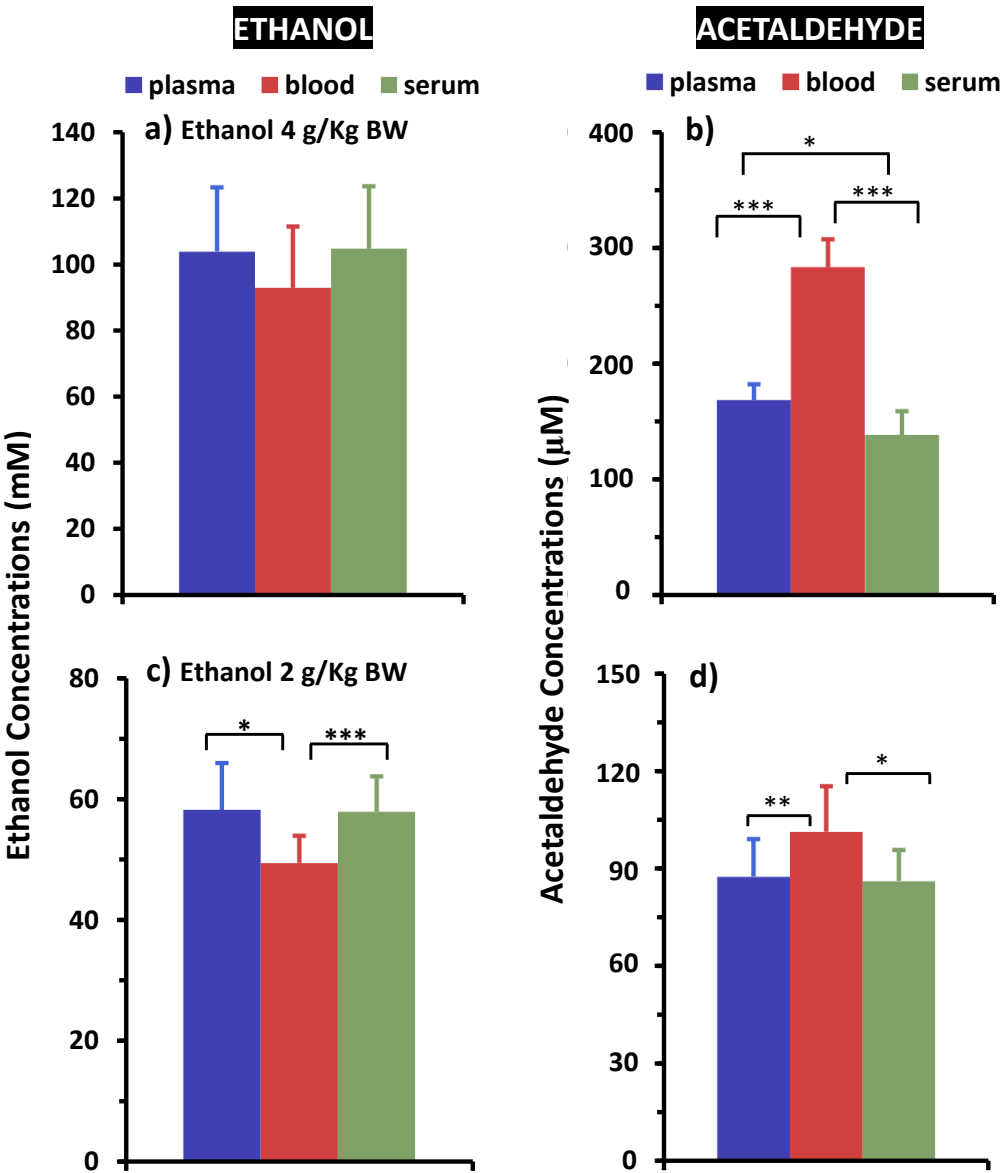

Supplement: Supplementary file 5 — Figure S5 [file ACER-49-1897-s005.pdf]
