# Supplementary material for: High‐throughput quantitation of acetaldehyde and ethanol in mice using gas chromatography/mass spectrometry positive chemical ionization
Source: Alcohol Clin Exp Res (Hoboken). 2025 Aug 4;49(9):1897–911. doi: 10.1111/acer.70126 (PMC12463767; doi:10.1111/acer.70126)
Supplement: Supplementary file 6 — Tables S1–S3 [file ACER-49-1897-s008.docx]

**Supplementary Material**

**Table 1**. Effects of thiourea (20 mM) on the acetaldehyde and ethanol concentrations in plasma samples (n = 6) of *Aldh2* KO mice given a single oral dosage of ethanol at 2 g/Kg BW.

| **Analytes** | | **Control (H_2_O)** | | | |  |  | | **Thiourea (20 mM)** | | | | | | | ***P* value** | | | |
| --- | --- | --- | --- | --- | --- | --- | --- | --- | --- | --- | --- | --- | --- | --- | --- | --- | --- | --- | --- |
|  |  | | Mean | | SD | CV(%) | | Mean | | | SD | | CV(%) | | | |  |  |  |
| ***Thiourea was added at the time of sample collection.*** | | | | | | | | | | | | | | |  | | | |  |
| [Ethanol], mM |  | | 57.4 | ± | 3.4 | 5.9 |  | | 56.7 | ± | | 2.9 | | 5.1 | | 0.698 | | | |
| [Acetaldehyde], µM | | 103.9 | | ± | 6.4 | 6.1 |  | | 99.7 | ± | | 5.7 | | 5.7 | | 0.256 | | | |
| ***Thiourea was added before the analyses after being stored at – 80 °C.*** | | | | | | | | | | | | | | |  | | | |  |
| [Ethanol], mM |  | | 54.4 | ± | 3.1 | 5.7 |  | | 55.7 | ± | | 2.3 | | 4.1 | | 0.389 | | | |
| [Acetaldehyde], µM | | 93.9 | | ± | 6.1 | 6.5 |  | | 93.6 | ± | | 4.4 | | 4.7 | | 0.909 | | | |
