# Supplementary material for: High‐throughput quantitation of acetaldehyde and ethanol in mice using gas chromatography/mass spectrometry positive chemical ionization
Source: Alcohol Clin Exp Res (Hoboken). 2025 Aug 4;49(9):1897–911. doi: 10.1111/acer.70126 (PMC12463767; doi:10.1111/acer.70126)
Supplement: Supplementary file 7 — Data S1 [file ACER-49-1897-s003.pdf]

INSTRUMENT CONTROL PARAMETERS:    8890-5977B
--------------------------------------------

D:\MassHunter\GCMS\1\methods\Acetaldehyde.M
Sat Sep 07 10:39:59 2024

Control Information
------- -----------

Sample Inlet : GC
Injection Source : Headspace
Injection Location: Front
Mass Spectrometer : Enabled

Headspace Parameters

Agilent 7697A
Temperature Settings:

| | |
|---|---|
| Oven Temperature | 60 °C |
| Loop Temperature | 70 °C |
| Transfer Line Temperature | 75 °C |

Timing Settings:

| | |
|---|---|
| Vial Equilibration | 10.00 min |
| Injection Duration | 0.50 min |
| GC Cycle Time | 15.00 min |

Vial and Loop Settings:

| | |
|---|---|
| Vial Size | 20 |
| Vial Shaking | Level 4, 50 shakes/min with acceleration of 180 cm/s² |
| Fill Mode | Default |
| Fill Flow | 50 |
| Fill Pressure | 15 psi |
| Pressure Equilibration Time | 0.1 |
| Loop Size | 1 mL |
| Loop Fill Mode | Custom |
| Loop Ramp Rate | 20 psi/min |
| Loop Final Pressure | 8 psi |
| Loop Equilibration Time | 0.05 |

Carrier Settings:

| | |
|---|---|
| Carrier Control Mode | GC controls Carrier |

Advanced Settings:

| | |
|---|---|
| Extraction Mode | Single Extraction |
| Vent After Extraction | OFF |
| Post Injection Purge | Default, 100 mL/min for 1 min |
| Acceptable Leak Check | Default, 0.2mL/min |

Sequence Actions:

| | |
|---|---|
| Vial Missing | Skip |
| Wrong Vial Size | Continue |
| Leak Detected | Continue |
| System Not Ready | Abort |

No Sample Prep method has been assigned to this method.

GC

GC Summary

| | |
|---|---|
| Run Time | 4.76 min |
| Post Run Time | 1 min |

Oven

Temperature

| | |
|---|---|
| Setpoint | On |
| (Initial) | 32 °C |
| Hold Time | 2.6 min |
| Post Run | 150 °C |
| Program | |
| #1 Rate | 50 °C/min |
| #1 Value | 60 °C |
| #1 Hold Time | 0 min |
| #2 Rate | 100 °C/min |
| #2 Value | 70 °C |
| #2 Hold Time | 1.5 min |

| | |
|---|---|
| Equilibration Time | 0 min |
| Max Temperature | 260 °C |
| Maximum Temperature Override | Disabled |
| Slow Fan | Disabled |

ALS

| | |
|---|---|
| Front Injector | Disabled |

Front SS Inlet He

| | |
|---|---|
| Mode | Split |
| Heater | On 220 °C |
| Pressure | On 7.6413 psi |
| Total Flow | On 216.06 mL/min |
| Septum Purge Flow | On 3 mL/min |
| Pre-Run Flow Test | Off |
| Gas Saver | On 20 mL/min after 3 min |
| Split Ratio | 200 :1 |
| Split Flow | 212 mL/min |
| Liner | Agilent 18740-80190: 800 µL (Split, straight, cup (manual injectio) |

Back SS Inlet He

| | |
|---|---|
| Excluded from Readiness | ***Excluded from Affecting GC's Readiness State*** |
| Mode | Split |
| Heater | Off |
| Pressure | Off |
| Total Flow | Off |
| Septum Purge Flow | Off |
| Pre-Run Flow Test | Off |
| Liner | A Liner has not been selected. |

Thermal Aux 2 (Transfer Line Interface)

Temperature

| | |
|---|---|
| Setpoint | On |
| (Initial) | 200 °C |

Column

Column #1

Flow

| | |
|---|---|
| Setpoint | On |
| (Initial) | 1.06 mL/min |
| Post Run | 2 mL/min |

| | |
|---|---|
| Description | DB-Select 624 Ultra Inert |
| Temperature Range | -20 °C—260 °C (260 °C) |
| Dimensions | 30 m x 250 µm x 1.4 µm (Uncalibrated) |
| Heater | Oven |
| Column lock | Unlocked |
| In | Front SS Inlet He |
| Out | MSD |
| (Initial) | 32 °C |
| Pressure | 7.6413 psi |
| Flow | 1.06 mL/min |
| Average Velocity | 37.179 cm/sec |

Holdup Time 1.3448 min
Control Mode Constant Flow

Column Outlet Pressure 0 psi

Detector Evaluation
Perform Detector Evaluation Test Off

MSD Transfer Line
Temperature
Setpoint On
(Initial) 250 °C

PCM A
PCM A He
Pressure
Setpoint Off
(Initial) 10 psi

Excluded from Readiness ***Excluded from Affecting GC's Readiness State***
PCM A He (unused)

Aux PCM A He
Pressure
Setpoint Off
(Initial) 10 psi

Excluded from Readiness ***Excluded from Affecting GC's Readiness State***
Control Mode Forward Pressure
Aux PCM A He (unused)

PSD 2
Pressure
Setpoint Off
(Initial) 10 psi
Post Run 10 psi

Excluded from Readiness ***Excluded from Affecting GC's Readiness State***
PSD Purge Off
PSD 2 He (unused)

MS Information
-- -----------

JetClean:  No Cleaning

General Information
------- -----------

Acquisition Mode         : SIM
Solvent Delay (minutes)  : 0.5
Tune file                : D:\MassHunter\GCMS\1\5977\pcich4250.u
EM Setting mode Gain     : 4.000000

Number of SIM Groups     : 1
Run Time (if MS only)    : 4.42 minutes

[SIM Parameters]
Group 1            Group ID               : 1
Resolution              : 1
Group Start Time         : 0.5
Number of Ions           : 5
Ions
Dwell In Group           :(  Mass,  Dwell) (  Mass,  Dwell)  (  Mass,  Dwell)
(  45.10,50  )  (  47.10,50  )
(  49.10,50  )  (  52.10,50  )

[MSZones]

MS Source                : 250 C   maximum 300 C
MS Quad                  : 150 C   maximum 200 C

Timed Events
----- ------
Number Events= 0

END OF MS ACQUISITION PARAMETERS

TUNE PARAMETERS for SN: US2236N001
---------------------------------

Trace Ion Detection is ON.

240.108 : EMISSION
94.964 : ENERGY
3.092 : REPELLER
134.898 : IONFOCUS
9.405 : ENTRANCE_LENS
1006.775 : EMVOLTS
1405.8 : Actual EMV
4.00 : GAIN FACTOR
2061.000 : AMUGAIN
140.188 : AMUOFFSET
1.000 : FILAMENT
1.000 : DCPOLARITY
19.607 : ENTLENSOFFSET
0.000 : Ion_Body
0.000 : EXTLENS
-218.000 : MASSGAIN
-15.209 : MASSOFFSET
CI Flow Rate: 23
CI A/B Gas : 1

END OF TUNE PARAMETERS
----------------------

END OF INSTRUMENT CONTROL PARAMETERS
------------------------------------
