# Supplementary material for: High‐throughput quantitation of acetaldehyde and ethanol in mice using gas chromatography/mass spectrometry positive chemical ionization
Source: Alcohol Clin Exp Res (Hoboken). 2025 Aug 4;49(9):1897–911. doi: 10.1111/acer.70126 (PMC12463767; doi:10.1111/acer.70126)
Supplement: Supplementary file 8 — Data S2 [file ACER-49-1897-s006.docx]

**Supplementary Material – Text**

**Materials & Methods / Sample Processing**

The Direct PCA Procedure applied in this method was developed from our previous conventional procedures, referred to as “ISTD Procedure,” in which ^2^H_5_-ethanol and ^2^H_4_-acetaldehyde were mixed with samples before PCA0.6N precipitated proteins. The Direct PCA Procedure simplified sample processing by diluting internal standards in PCA0.6N as solution S0102 and then adding S0102 to each sample. It greatly shortened the sample processing time, minimized the possible errors yielded from the high volatility of acetaldehyde and non-specific conversion from ethanol to acetaldehyde, and was labor-saving.

The simplified Direct PCA Procedure was compared with the ISTD Procedure in a pooled sample collected from WT mice given orally 4 g/Kg BW of ethanol. Calibration curves were constructed independently in mouse plasma as a matrix. Compared to the regression equations from calibration curves (Figure 2) using the Direct PCA Procedure, those applying the ISTD Procedure were slightly different. The latter were y = 0.886x + 0.0023 for ethanol, R^2^ = 0.9999, and y = 0.9448x + 0.0355 for acetaldehyde, R^2^ = 0.9996. The slope of the calibration curve using the Direct PCA Procedure was greater for ethanol (*a* = 1.066) but similar for acetaldehyde (*a* = 0.9417).

Based on the corresponding calibration curves, serum acetaldehyde and ethanol concentrations (n = 6) were 38.0 ± 4.7 µM and 106.6 ± 4.7 mM using the Direct PCA Procedure; and 33.5 ± 4.5 µM and 116.7 ± 7.1 mM using the ISTD Procedure. Significant differences were observed in ethanol concentrations (*P* = 0.033) but not in acetaldehyde concentrations (*P* = 0.078). The percentage of differences between the concentrations determined using two procedures was 12.1% for acetaldehyde and 9.1% for ethanol.

In summary, acetaldehyde and ethanol concentrations differed when the two procedures were applied. However, after method validation, the Direct PCA Procedure proved to be compatible and reliable for quantitating acetaldehyde and ethanol in rodent samples at high throughput.

**Discussion / Appearance and disappearance of ethanol in mouse circulation**

The observations of the ethanol time-course shared some similarities and presented differences with previous studies (**Pruett et al., 2020**). When given a similar oral dosage of ethanol, about 4 g/Kg BW, the observed peak time for ethanol was similar to that reported by Carson and Pruett (**Carson and Pruett, 1996**) in the tail blood from B6C3F1 female mice, but 30 min later than that reported by Livy and coauthors in serum collected from the decapitation of C57BL/6J mice (**Livy et al., 2003**). However, greater differences were observed in the ethanol concentrations. The above studies presented lower maximal concentrations (50 – 60 mM) than those in the current study (86 – 109 mM) and much shorter half-lives, in which the ethanol disappeared at 4 - 5 hr. Furthermore, the acetaldehyde was not detected in either of the studies (**Pruett et al., 2020**), nor was the comparison of ethanol concentrations in various blood forms conducted. In contrast, our study observed that the ethanol concentrations appeared high around 60 mM at 6 hr, consistent with our prior study (**Mackowiak et al., 2022**), in addition to the comparison of both acetaldehyde and ethanol in mouse plasma, whole blood, and serum.

**Discussion / Sex differences**

The data presented in this study have shown that acetaldehyde levels were higher in erythrocytes than in plasma, suggesting that hemoglobin in erythrocytes might play an important role in the elevated acetaldehyde concentration observed in whole blood, probably via forming acetaldehyde-hemoglobin adducts. According to Frith et al. (**Frith et al., 1980**), there are no significant differences in the hemoglobin concentrations in male and female mice at varied ages. Therefore, while acetaldehyde concentrations may differ between the sexes, the observed pattern of higher acetaldehyde levels in whole blood compared to serum or plasma is probably consistent across both sexes.

**References**

CARSON, E. J. & PRUETT, S. B. 1996. Development and characterization of a binge drinking model in mice for evaluation of the immunological effects of ethanol. *Alcohol Clin Exp Res,* 20**,** 132-8.

FRITH, C. H., SUBER, R. L. & UMHOLTZ, R. 1980. Hematologic and clinical chemistry findings in control BALB/c and C57BL/6 mice. *Lab Anim Sci,* 30**,** 835-40.

LIVY, D. J., PARNELL, S. E. & WEST, J. R. 2003. Blood ethanol concentration profiles: a comparison between rats and mice. *Alcohol,* 29**,** 165-71.

MACKOWIAK, B., XU, M., LIN, Y., GUAN, Y., SEO, W., REN, R., FENG, D., JONES, J. W., WANG, H. & GAO, B. 2022. Hepatic CYP2B10 is highly induced by binge ethanol and contributes to acute-on-chronic alcohol-induced liver injury. *Alcohol Clin Exp Res,* 46**,** 2163-2176.

PRUETT, S., TAN, W., HOWELL, G. E., 3RD & NANDURI, B. 2020. Dosage scaling of alcohol in binge exposure models in mice: An empirical assessment of the relationship between dose, alcohol exposure, and peak blood concentrations in humans and mice. *Alcohol,* 89**,** 9-17.
